# Disadvantageous Socioeconomic Position at Specific Life Periods May Contribute to Prostate Cancer Risk and Aggressiveness

**DOI:** 10.3389/fonc.2018.00515

**Published:** 2018-11-15

**Authors:** Sreenath Madathil, Christine Blaser, Belinda Nicolau, Hugues Richard, Marie-Élise Parent

**Affiliations:** ^1^Epidemiology and Biostatistics Unit, INRS-Institut Armand-Frappier, Université du Québec, Laval, QC, Canada; ^2^Division of Oral Health and Society, Faculty of Dentistry, McGill University, Montreal, QC, Canada; ^3^School of Public Health, Université de Montréal, Montreal, QC, Canada

**Keywords:** life course, prostate cancer, socioeconomic position, childhood, adolescence, occupation, Bayesian relevant life course exposure model

## Abstract

**Background:** Previous studies on socioeconomic position (SEP) and risk of prostate cancer (PCa) have produced contradictory results. Most measured SEP only once during the individuals' life span. The aim of the study was to identify life course models that describe best the relationship between SEP measured during childhood/adolescence, early- and late-adulthood, and risk of PCa overall as well as according to tumor aggressiveness at diagnosis.

**Methods:** We used data from a population-based case-control study of PCa conducted in the predominantly French-speaking population in Montreal, Canada. Cases (*n* = 1,930) with new, histologically-confirmed PCa were ascertained across hospitals deserving the French-speaking population in 2005–2009. Controls (*n* = 1,991), selected from Quebec's list of French-speaking electors, were frequency-matched to cases (±5 years). In-person interviews collected information on socio-demographic and lifestyle characteristics, and a complete occupational history. Measures of SEP during childhood/adolescence included parents' ownership of a car and father's longest occupation, while the subject's first and longest occupations were used to indicate early- and late-adulthood SEP, respectively. We used the Bayesian relevant life course exposure model to investigate the relationship between lifelong SEP and PCa risk.

**Results:** Cumulative exposure to disadvantageous SEP was associated with about a 50% increase in odds of developing PCa. Late-adulthood SEP was identified as a sensitive period for aggressive PCa. Childhood/adolescence SEP based on parents' ownership of a car was associated with non-aggressive PCa. Associations were independent from PCa screening.

**Conclusion:** Disadvantageous SEP over the life course was associated with higher PCa incidence, with consistent evidence of sensitive time periods for cancer aggressiveness. The mechanisms through which disadvantageous SEP relates to PCa risk need to be further elucidated.

## Introduction

In Canada, one out of five newly detected cancers in 2017 were prostate cancers (PCa), making these the most common solid-tumor cancer among men (1). It was also the third cause of male cancer-related deaths. A few non-modifiable risk factors such as age, family history of PCa, and ancestry have been established (2). Regarding the latter, data from the United-States indicate striking racial/ethnic differences in PCa incidence and mortality rates (3) with American Asian/Pacific Islanders having the lowest incidence of PCa, followed by whites and African-Americans who experience the highest burden of the disease (4). These racial/ethnic disparities are not well-understood, but it is believed that they result from the interplay between biological, environmental, and social risk factors in cancer initiation (5, 6).

Socioeconomic position (SEP) (7) represents an umbrella of factors that may collectively influence the burden of PCa, including behavioral and environmental risk factors as well as access to health care (e.g., cancer screening). There is mounting evidence that a favorable SEP is associated with better PCa survival (8–10). However, the relationship between SEP and PCa incidence is much less clear, with studies documenting positive, negative, or no associations (11–24), even when the timing of introduction of the prostate specific antigen (PSA) test is taken onto account. These inconsistencies might be explained by the fact that most of the work investigating this association assessed SEP only once during the individual's life span usually in adult life, potentially overlooking other relevant exposure periods.

Studies of the developmental origins of adult health and disease have produced evidence highlighting the importance of prenatal and infant life (25). They led to the understanding that exposure to insults during rapid organ system development is critical to adult health. The prostate gland is essentially dormant until puberty, when a complex interaction between sex and other growth hormones induces rapid development (26, 27). Studies examining pre-adult exposures and risk of PCa remain rare (28).

Life course epidemiology (29) offers a framework to further our understanding of the long term effects of SEP on PCa risk. The theory can be operationalized under three main models: (i) accumulation, in which exposures throughout life are equally important and their effects accumulate over time; (ii) critical period, in which exposures during a specific period in life lead to irreversible effects regardless of exposures in other periods; and (iii) sensitive period, in which exposure during one or more periods have a higher effect compared to the others.

Important insights into PCa etiology may be gained by identifying which life course model describes best the role of SEP in cancer risk. For example, adolescence is a time of rapid and profound change in hormone levels and in body composition, entailing the development of secondary sexual characteristics and achievement of fertility (30). It is possible that early-life SEP shapes the environment during this important developmental window of vulnerability, increasing the risk of PCa.

Moreover, exposures in specific periods may be hypothesized to affect tumor aggressiveness. Aggressive and non-aggressive PCa appear to have different sets of anthropometric (31), lifestyle (32) and occupational (33) risk factors, supporting the notion that their etiology might be under distinct influences. Recent observations also indicate that grade is established early in prostate tumor pathogenesis (34). This raises the possibility that aggressive cancers represent a specific etiological entity, possibly developing under exogenous influences that operate at a specific time over the life course.

In this paper, we identify the life course models that describe best the relationship between life course SEP measured during childhood/adolescence, early- and late-adulthood and the risk of incident PCa overall as well as according to tumor aggressiveness at diagnosis.

## Materials and methods

### Study population and data collection

#### Study population

The Prostate Cancer & Environment Study (PROtEuS), set in Montreal, Canada, has been described previously (32, 35). In order to ensure comprehensive population coverage at recruitment and comply with institutional regulations, the study base was restricted to men who referred or would be expected to refer to a French hospital for a PCa diagnosis. This represents the vast majority of Montreal residents, as more than 75% of them speak French at home (36). Cases (*n* = 1,930), aged ≤75 years, newly diagnosed with histologically-confirmed PCa (ICD-10, code C61), were identified from pathology departments across seven French hospitals between September 1, 2005 and December 31, 2009. Comparison with the provincial tumor registry indicates that these represented over 80% of all incident PCa cases in the area. Control subjects (*n* = 1,991) were recruited concurrently from Quebec's continually updated electoral list of French-speaking individuals. The electoral list is considered to include nearly all Canadian citizens living in Quebec. Controls were randomly selected from an area comprising 39 electoral districts, corresponding to those of the cases, and frequency-matched by 5-year age groups. Participation rates among cases and controls were 79 and 56%, respectively. Reasons for non-participation, among cases and controls, were refusal (94 and 86%, respectively), unable to trace (3 and 11%), death with no proxy available (2 and 1%), language barrier (1 and 1%) or too sick to participate (1% of controls). Ethics committees of all participating hospitals approved the study. All participants provided written informed consent.

#### Data collection

As part of in-person interviews, specially trained interviewers collected data on a wide range of exposures including socio-demographic characteristics, lifestyle (physical activity level, smoking, alcohol consumption, body mass index, etc.) and a detailed occupational history for all jobs held throughout the lifetime. Gleason scores were extracted from pathology reports.

#### Measurement of life course SEP

Three periods in life were considered to compile the SEP variables from collected data: childhood/adolescence, early-adulthood (first entry into job market) and late-adulthood.

Childhood/adolescence SEP was assessed using two different indices. The first consisted of participants' report of parents' ownership of a car (yes/no) when they were younger than 16 years old. Those who did not have car access were assigned a disadvantageous SEP (coded 1), while others were classified in the advantageous SEP category (coded 0). The second index used was the longest occupation held by the participant's father, which was coded according to the 1988 International Standard Classification of Occupations (37), then assigned a binary SEP level (described below).

SEP during early- and late-adulthood periods were assessed using the first and longest occupation held by the participant, respectively. Careful evaluation of job descriptions and additional information about employment was conducted to assign a job title to each occupation lasting 1 year or more. Occupations were coded according to the four-digit 1988 International Standard Classification of Occupations (37). These codes were collapsed into 10 categories of increasingly disadvantageous SEP according to the European Socioeconomic Classification (ESeC) (38). We used the dichotomized version of advantageous /disadvantageous SEP in our analyses where “lower services” (category 8), “lower technical”(category 9) and “routine” (category 10) were grouped as disadvantageous SEP (coded 1) while categories 1–7 fell into the advantageous SEP (coded 0) group.

### Statistical analyses

#### Study sample

There were 3,921 subjects (1,930 cases and 1,991 controls) available for analyses. However, some of these were excluded owing to missing values for some of the SEP indicators, i.e., 20 (0.5%) for parent's car ownership and 133 (3%) for the fathers' longest occupation. Missing values for covariates (ranging from 0 to 2.9%) were assigned to a missing indicator category.

#### Bayesian model for life course investigation

**Figure 1:**
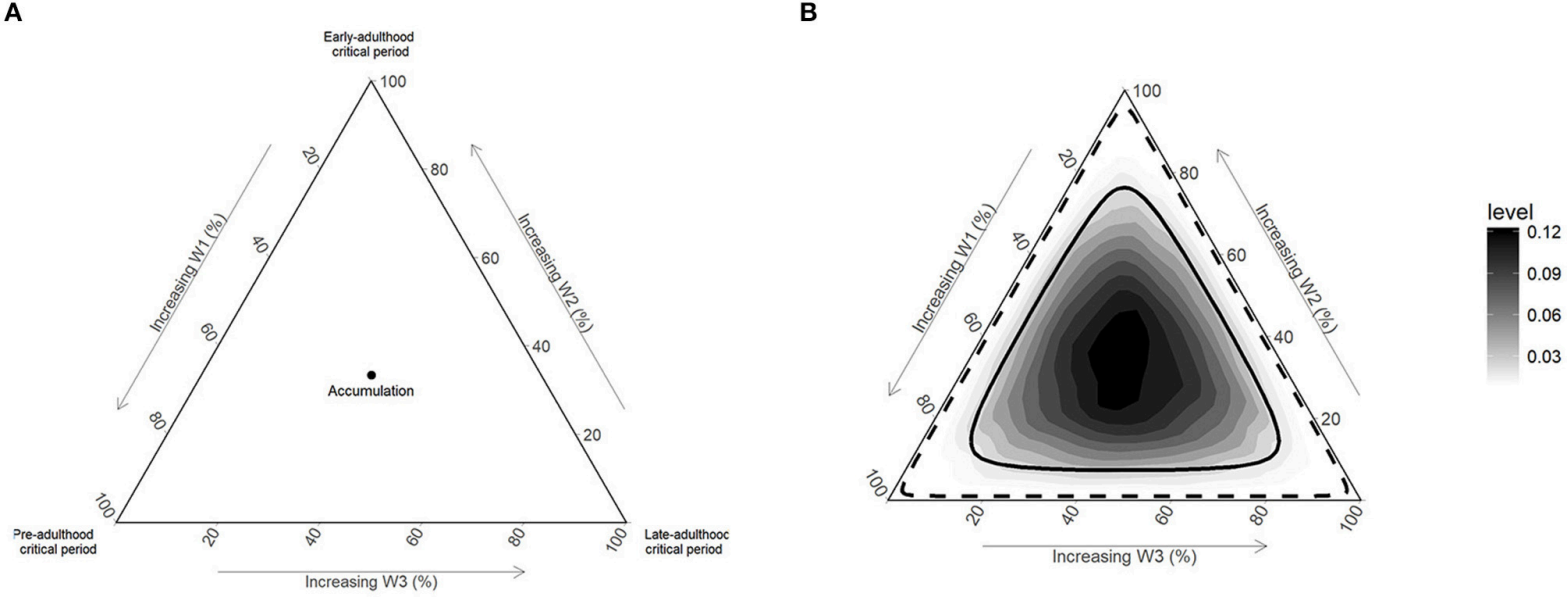
Illustration of life course hypotheses and non-informative prior distribution on ternary plots. **(A)** Vertices of ternary plot represents critical period hypotheses, center points represent the accumulation hypothesis and other point in the plot represent sensitive period hypothesis; **(B)** Dirichlet (1, 1, 1) prior with 50% (solid line) and 95% (dashed line) credible intervals.

We recently proposed a Bayesian relevant life course exposure model (BRLM) to identify periods of life in which exposure have the highest impact on the outcome (39). Although we used continuous exposures to demonstrate the technique, it can be used for binary exposures. Briefly, the model assumes weights for exposures occurring at different periods of life and an overall effect for the lifetime exposure. Thus, it allows exposures to contribute differently to the disease process depending on the life period when it happened and also allows the accumulation of these effects temporally. The values estimated for the weights jointly provide information on the life course hypothesis supported by the data. For three life periods, as in our study, the joint distribution of estimates can be visualized using a ternary plot (Figure 1A). The vertices represent critical period hypotheses and the central point represents the accumulation hypothesis. Distributions with higher densities at areas not close to these points represent the sensitive period hypothesis. Each side in the ternary plot represents the weights corresponding to one life period.

The joint posterior distribution of weights needs to be interpreted together with the overall effect. The weights represent the relative importance of being in a disadvantageous SEP during different life periods and provide information on the life course hypothesis supported by the data. The overall effect represents the effect of being in a disadvantageous compared to advantageous SEP during all life periods. This effect is equivalent to the maximum accumulated effect of SEP over the life course. An alternative way of inference is to compute period specific effects. These estimates represent the time dependent association between the exposure and outcome. The period specific estimates are combination of overall effect and relative weights estimated from the BRLM.

We used the BRLM, with unconditional logistic regression likelihood, to identify the period (childhood/adolescence, early-adulthood, and late-adulthood) during participants' lives that is most sensitive to disadvantageous SEP exposure in relation to the risk PCa later in life.

The BRLM provides the opportunity to transparently include prior beliefs about life course hypotheses in the analysis. However, in case of little or no prior evidence in the field, a non-informative prior can be used to allow the data to drive the results; such a prior will give equal weak support to all life course hypotheses. We used a non-informative Dirichlet (1, 1, 1) prior for the weights (Figure 1B) and a weakly informative student_t (3,0,2.5) prior for other regression parameters. Mean and 95% credible intervals (95%Crl) for weights and odds ratios (OR) for the overall effect were computed from their corresponding posterior distributions.

PCa screening is a strong determinant of cancer diagnosis. Inclusion of latent, undetected PCa in the control series would lead to an attenuation of risk estimates. Moreover, screening can be related to SEP, thereby confounding associations. In order to rule out the role of screening practices, we restricted the analyses to participants who were screened at least once for PCa in the previous 2 years.

Further, to investigate the association between disadvantageous SEP and PCa aggressiveness, we conducted analyses stratifying the cases according to cancer grade. Tumors with Gleason scores <7 were defined as non-aggressive, while scores ≥7 designated aggressive tumors (40).

Main analyses were adjusted for age (continuous), ancestry (categorical) and family history of PCa (yes, no), cigarette smoking (ever, never), alcohol consumption (drink-years), physical activity (not very active, moderately active, very active), and body mass index (BMI) 2 years prior to the diagnosis or interview (continuous).

#### Sensitivity analyses

We conducted a series of sensitivity analyses to explore different dimensions of our findings. We refit all our models replacing the variable on parental car ownership by the longest occupation held by the father of the participant. In addition to being a common measure of SEP during childhood and adolescence, the use of this occupational variable standardizes the indicator of SEP across the life course.

To assess the impact of restricting participants to men who were recently screened, we conducted a sensitivity analysis including all participants regardless of their screening status. Further, to assess the influence of lifestyle factors on the association between SEP and PCa, we compared the results from the full model used in the main analyses with those from a reduced model in which only age, ancestry, and family history of PCa were considered as potential confounders.

All analyses were performed using the R package to fit Bayesian models using Stan (41, 42).

## Results

Selected characteristics of the 1,930 cases and 1,991 controls are presented in Table 1. Controls were about 1 year older, on average, than cases, reflecting the slightly longer time needed to recruit controls. As expected, cases presented a significantly higher proportion of first-degree family members with PCa, had a higher proportion of participants of African ancestry, and a lower proportion of Asians than controls. No major differences between the groups were observed in relation to cumulative use of alcohol and smoking status as well as physical activity levels. Nearly all cases and 76% of controls had been screened in the 2 years prior to diagnosis/interview. The Gleason score was missing for three participants. The proportion of participants with at least one first-degree relative with PCa was higher among non-aggressive cases compared to aggressive ones.

**Table 1:** Selected characteristics of study participants.

	**Controls** **(*n* = 1,991)**	**Cases^a^**		
		**All cancers** **(*n* = 1,930)**	**Non-aggressive cancers** **(*n* = 1,376)**	**Aggressive cancers** **(*n* = 532)**
**Age (Mean** ± **SD)**	64.84 ± 6.88	63.56 ± 6.80	63.17 ± 6.83	64.61 ± 6.61
**Ancestry**, ***n*** **(%)**				
African	87 (4.4)	126 (6.5)	95 (6.8)	31 (5.8)
Asian	67 (3.4)	22 (1.1)	14 (1.0)	8 (1.5)
European	1,649 (82.8)	1,654 (85.7)	1,196 (85.9)	455 (85.0)
Other	174 (8.7)	116 (6.0)	80 (5.7)	36 (6.7)
Do not know	14 (0.7)	12 (0.6)	7 (0.5)	5 (0.9)
**Timing of last prostate cancer screening**, ***n*** **(%)**				
Never	191 (9.6)	3 (0.2)	2 (0.1)	1 (0.2)
Within last 2 years	1,509 (75.8)	1,910 (99.0)	1,375 (98.8)	532 (99.4)
More than 2 years ago	234 (11.8)	1 (0.1)	1 (0.1)	0 (0.0)
Do not know	57 (2.9)	16 (0.8)	14 (1.0)	2 (0.4)
**Cigarette smoking**, ***n*** **(%)**				
Never	514 (25.8)	515 (26.7)	386 (27.7)	129 (24.1)
Ever	1,476 (74.1)	1,414 (73.3)	1,006 (72.3)	405 (75.7)
Do not know	1 (0.1)	1 (0.1)	0 (0.0)	1 (0.2)
**Alcohol use**, ***n*** **(%)**				
Never	231 (11.6)	210 (10.9)	154 (11.1)	56 (10.5)
Ever	1759 (88.3)	1718 (89.0)	1237 (88.9)	478 (89.3)
Do not know	1 (0.1)	2 (0.1)	1 (0.1)	1 (0.2)
**Physical activity**, ***n*** **(%)**				
Not very active	486 (24.4)	435 (22.5)	327 (23.5)	107 (20.0)
Moderately active	558 (28.0)	524 (27.2)	383 (27.5)	140 (26.2)
Very active	946 (47.5)	971 (50.3)	682 (49.0)	288 (53.8)
Do not know	1 (0.1)	0 (0.0)	0 (0.0)	0 (0.0)
BMI (Mean ± SD)	27.18± 4.43	26.75 ± 4.01	26.72 ± 3.92	26.83 ± 4.24
**First-degree family history of prostate cancer**, ***n*** **(%)**				
No	1,737 (87.2)	1,414 (73.3)	1,002 (72.0)	411 (76.8)
Yes	198 (9.9)	450 (23.3)	344 (24.7)	105 (19.6)
Do not know	56 (2.8)	66 (3.4)	46 (3.3)	19 (3.6)

a *Low-grade (Gleason <7) defined as non-aggressive cancer; high-grade (Gleason ≥7) defined as aggressive cancer*.

**Figure 2:**
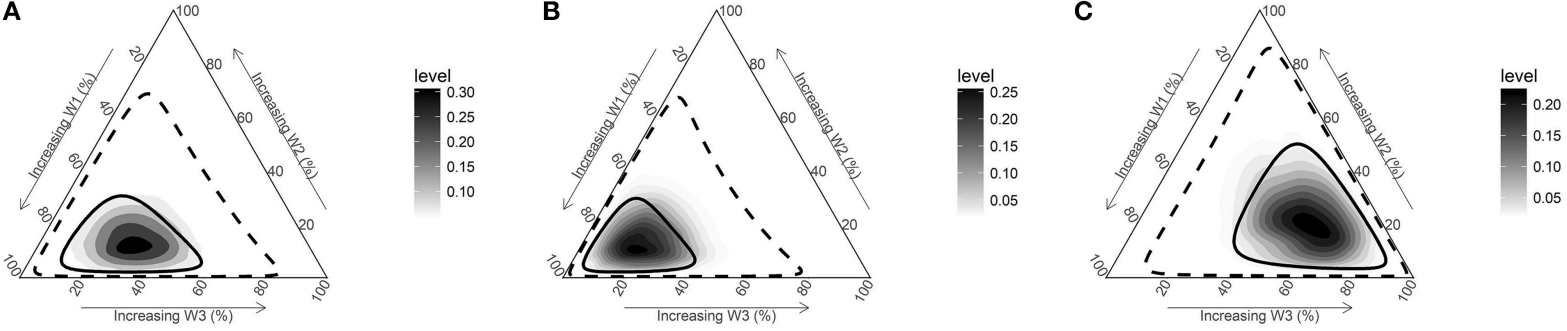
Posterior joint distribution of weights (w) estimated for three periods (childhood & adolescence based on parents' ownership of a car [w1], early adulthood [w2], late adulthood [w3]). **(A)** All PCa cases vs. control. **(B)** Non-aggressive PCa cases (Gleason <7) vs. controls. **(C)** Aggressive PCa cases (Gleason≥7) vs. controls. Solid and dashed line represents 50% and 95% credible intervals and darker areas represent higher posterior densities.

Being in a disadvantageous compared to an advantageous SEP during all three periods (childhood/adolescence, early adulthood, and late adulthood) increased the odds for overall PCa by 49% (OR = 1.49; 95%Crl = 1.20–1.83) (Table 2 and Figure 2A). There was evidence for a sensitive period of exposure to disadvantageous SEP in early life (childhood/adolescence period) for the risk of PCa (posterior probability 83.7%). Disadvantageous SEP in childhood/adolescence increased the odds of PCa by 26% (OR = 1.26; 95%Crl = 1.06–1.48) (Supplementary Table 1).

**Table 2:** Association between a disadvantageous socioeconomic position and prostate cancer risk.

	**Controls *n* (%)**	**Cases *n* (%)**	**OR (95% Crl)*^*a*^***	**Mean weight (95% Crl)**
**Any type of PCa**	**(*****n*** = **1,438)**	**(*****n*** = **1,773)**	
**Overall effects**			1.49 (1.20 - 1.83)
**Weights***^*b*^*			
Childhood & adolescence (w1)	677 (47.1)	845 (47.7)		0.59 (0.25–0.87)
Early Adulthood (w2)	839 (58.3)	1064 (60.0)		0.12 (0.00–0.37)
Late Adulthood (w3)	657 (45.7)	876 (49.4)		0.29 (0.03–0.63)
Posterior probability for an early-life sensitive period hypothesis (w1 > w2 & w3) = 83.7%				
**Non-aggressive PCa**	**(*****n*** = **1,438)**	**(*****n*** = **1,282)**	
**Overall effects**			1.48 (1.17 - 1.84)
**Weights**			
Childhood & adolescence (w1)	677 (47.1)	612 (47.7)		0.71 (0.38–0.94)
Early Adulthood (w2)	839 (58.3)	750 (58.5)		0.12 (0.01–0.36)
Late Adulthood (w3)	657 (45.7)	604 (47.1)		0.17 (0.01–0.47)
Posterior probability for an early-life sensitive period hypothesis (w1 > w2 & w3) = 95.9%				
**Aggressive PCa**	**(*****n*** = **1,438)**	**(*****n*** = **489)**	
**Overall effects**			1.52 (1.11 - 2.04)
**Weights**			
Childhood & adolescence (w1)	677 (47.1)	232 (47.4)		0.22 (0.01–0.59)
Early Adulthood (w2)	839 (58.3)	312 (63.8)		0.20 (0.01–0.60)
Late Adulthood (w3)	657 (45.7)	271 (55.4)		0.58 (0.12–0.92)
Posterior probability for a late-life sensitive period hypothesis (w3 > w1 & w2) = 79.6%				

a OR, Odds ratio; CrI, Credible interval. Model adjusted for age, ancestry, family history of PCa, body mass index, physical activity, cigarette smoking and alcohol drinking.

b *w1, weight 1; w2, weight 2; w3, weight 3. Weight 1 (childhood & adolescence) based on parents' ownership of a car, weight 2 (early adulthood) based on first occupation, weight 3 (late adulthood) based on longest occupation*.

*n, Number of study subjects*.

When we conducted analyses stratifying by PCa aggressiveness, we observed a similar pattern of association for non-aggressive PCa. Being in a disadvantageous compared to an advantageous SEP during all three periods increased the odds for PCa by 48% (OR = 1.48; 95%Crl = 1.17–1.84) (Table 2 and Figure 2B). Also, there was evidence for a sensitive period early in life in the relationship between disadvantageous SEP and non-aggressive PCa (posterior probability 95.9%). Being in a disadvantageous SEP during childhood/adolescence increased the odds of PCa by 32% (OR = 1.32; 95%Crl = 1.08–1.58) (Supplementary Table 1).

Although disadvantageous SEP during all three life periods increased the odds of aggressive PCa by 52% (OR = 1.52; 95%Crl = 1.11–2.04), results support a sensitive period in late adulthood (posterior probability 79.6%) (Table 2 and Figure 2C). Being in a disadvantageous SEP during late adulthood increased the odds of aggressive PCa by 28% (OR = 1.28 95%Crl = 1.02–1.63) (Supplementary Table 1).

Results from our sensitivity analysis replacing the childhood/adolescence SEP indicator parental car ownership by the father's longest occupation are presented in the Supplementary Tables 2, 3 and Supplementary Figures 1A–C. Although the overall direction of associations was similar, point estimates were attenuated when using the father's occupation, especially for non-aggressive PCa (Supplementary Table 2). Furthermore, the joint posterior distribution of weights for non-aggressive PCa showed no considerable difference from the non-informative prior distribution (Figure 1B, Supplementary Figure 1B), indicating a lack of information in the data to disentangle the life course hypotheses when using the father's occupation.

Including lifestyle covariates in our models tended to attenuate associations, but only marginally.

The results of sensitivity analyses including all participants regardless of screening for PCa showed agreement with main results for the direction of association, with weaker risk estimates. However, given the wide credible intervals, the results are inconclusive (Supplementary Tables 2, 3).

All other sensitivity analyses showed no considerable differences in direction or point estimate for the association compared to the main analyses.

## Discussion

To the best of our knowledge, this is the first large case-control study applying the life course epidemiology framework to investigate the association between life course SEP and risk of PCa.

We observed a positive association between disadvantageous SEP when considering together childhood/adolescence, early- and -late adulthood, and PCa. The overall effect of disadvantageous SEP showed a ≈50% increase in odds of the disease. The estimates demonstrated the cumulative nature of the effects; the trajectory with disadvantageous SEP during all three periods showed the strongest association with risk of PCa. In addition, we consistently identified the late-adulthood period as sensitive for aggressive PCa, independently from screening. Childhood/adolescence SEP was related to risk of non-aggressive PCa when using parental car ownership as indicator.

Previous studies have shown contradictory results on the association between adult SEP and incident PCa. Positive associations (10–14, 18, 19, 21), inverse associations (15, 20, 22, 24) or no association (23) have indeed been observed. Inconsistent results have also been reported across studies that considered the period of introduction of PSA testing (7, 43–46). Potential reasons behind these inconsistencies may relate to the assessment of SEP only once during the life course or to the use of different indicator variables for SEP.

There is no single best indicator of SEP suitable for all study aims and applicable at all-time points in all settings (7, 47). Each indicator measures different, often related aspects of socioeconomic stratification. For childhood/adolescence SEP, we performed a sensitivity analysis using two indicators, namely parents' ownership of a car and the father's longest occupation. While the latter is one of the most widely used SEP indicator for early life (43), car ownership, a marker of material living standards (7, 37), has been shown to be a useful indicator of childhood SEP in older adults in contemporary developed country populations (7, 44, 45). The indices we used were weakly correlated with one another, further suggesting that these may measure different constructs of SEP (Supplementary Table 4). One reason that might also explain the difference in our findings when using different SEP indicators during childhood/adolescence is measurement error. More subjects could not report their father's occupation, as compared to parental car ownership, potentially reflecting greater recall issues and thus measurement error.

In our study, when we replaced the indicator variable parents' ownership of a car for the father's longest occupation, results became largely inconclusive. Interestingly, a life course investigation on SEP and nine cancer types using data from a study conducted in Montreal also supports early life as sensitive period for PCa (any type), this time using the father's main occupation (48). However, the multi-site study was conducted approximately 25 years before PROtEuS, which may indicate that car ownership is a more sensitive measure of SEP in the more recent era, when the current study was conducted.

We elected to use the subjects' occupations from the lifetime work histories as indicators of early and late-adulthood SEP (7). A major focus of our main study is to evaluate the role of occupational exposures in PCa. To this end, we collected a detailed description of all jobs held over the lifetime, which is something other studies rarely have. This is important in the context of evaluating SEP at specific time periods such as here. With increasing interest in the role of SEP across the life course, other studies have used individuals' occupations at different stages in adult life (46). We used the first occupation to represent SEP in early adulthood, and the longest occupation to represent late-adulthood SEP. This choice was motivated by the mean age at the beginning and end of these jobs. In our data, the mean age of participants at beginning of their first job was 20 ± 4 years and at end of first job it was 27 ± 11 years (mean duration = 8 ± 10 years). Corresponding values for the longest job were 33 ± 10 years and 55 ± 10 years (mean duration = 22 ± 9 years), respectively. The correlation between the two occupations used was moderate, at 0.55, indicating that only about 30% of the variance in SEP based on one occupation explained the variation in the other. This provided us with the ability to assign SEP at the two time points of interest.

Studies observing increased risk among advantageous SEP groups attributed such finding to greater medical attention, lifestyle (13, 49) and access to PSA screening (12, 21). Our study was set in Montreal, where the population has free, universal access to health care and where, at the time the study was conducted, PCa screening was often part of the yearly routine exam. Screening uptake was high in our analytical sample with 76% of controls having been screened for PCa in the 2 years preceding the interview. This reduces the likelihood that PCa detection would be an important factor underlying our findings. Of note, associations went in the same direction, albeit stronger, when restricting analyses to men recently screened.

In addition, our analysis took into consideration PCa aggressiveness, defined by tumor grade. Gleason's grade describes tumor cell differentiation patterns, with higher-grade tumors being more aggressive and having a poorer prognosis (34); it does not reflect disease progression (50). Few previous studies have distinguished localized and aggressive PCa or cancer stage (11, 13, 20, 22). Our findings suggest that the effects of SEP were different for low- and high-grade cancers. This observation could be interpreted as indicative of the existence of two types of cancer with different etiologies, which has also been proposed by others (13). Recent findings indeed suggest that PCa grade may be established early in tumor pathogenesis and that Gleason grade progression is uncommon (34). Low-grade PCa has been shown to diverge early from high-grade PCa and there appears to be no direct progression from low-grade to metastatic disease (51).

The effect of disadvantageous SEP in childhood/adolescence suggested by our findings could be attributable to several factors. For example, a poor diet can disturb the pre-adulthood hormonal milieu (52) at the time when the prostate develops most quickly. This is reflected in lower height (53) and childhood obesity (54). Similarly, disadvantageous SEP in early years could lead to more stress, which is also known to negatively affect growth and development (55). Although previous studies have examined the effect of adiposity (27) and energy restriction during adolescence as well as height and weight (27, 56, 57) on PCa risk, results are conflicting.

Adult SEP may also act through common risk factors such as health behaviors (e.g., tobacco smoking and excessive alcohol consumption) or obesity, which are more prevalent among individuals with disadvantageous SEP (58). However, adjustment for these factors had a modest impact on results. There are as yet very few confirmed risk factors for PCa, leaving uncertainty about the covariates to be adjusted for when studying PCa risk. While our analysis took into account a number of health behaviors, residual confounding or lack of adjustment for factors not yet recognized in PCa development cannot be ruled out. Disadvantageous SEP during adulthood may be linked to occupational features associated with PCa such as shift work (59) and exposure to chemical agents (33, 60). Further adjustment for these factors did not alter findings (data not shown). SEP is a complex construct that represents an array of combined exposures and it may not accurately represent specific risk factors. Conversely, it may be that these factors act differently in puberty and adulthood.

The role of life course SEP in PCa risk has not been studied extensively. The mechanisms underlying the associations observed are not known. Yearly PCa screenings were common in this study population and associations with SEP were even stronger in analyses restricted to men recently screened, suggesting that screening does not explain the SEP-PCa relationship in our study base. It also appears that the lifestyle factors we considered were not major explanatory factors. Hypothetically, some factors unaccounted for and influencing cancer promotion, possibly diet, could underlie the late-adulthood SEP-aggressive PCa association.

There are several limitations that need to be considered when evaluating this work. The first relates to the misclassification of exposure to SEP. Although we used several SEP indicators, these might not capture the full spectrum of this construct. Variables were based on self-reports, which likely entailed errors, possibly more so among older subjects. Our age limit of 75 years for study participation may have helped alleviate age-related reporting errors to a certain extent. However, it is likely that reporting errors affected cases and controls in a similar fashion, thus keeping the risk estimates closer to unity. We conducted a sensitivity analysis excluding proxy respondents, who might be less cognizant of subjects' exposures, and observed no difference in results. Nevertheless, occupational circumstances are a specific focus of the PROtEuS study and our team has considerable experience in eliciting detailed work histories and in assigning occupational titles (33, 61). Moreover, reports of work histories have been shown to be valid (62). The commonly used ISCO 1988 and ESec were applied to classify occupations and assign SEP. Car ownership by parents may have different meanings, although in the era when subjects were growing up, it probably was a reasonable indicator of affluence. SEP was dichotomized in a crude fashion into advantageous and disadvantageous, which may have masked differentials in exposure. While there was overlap in the coverage of SEP assessments in adulthood for some subjects, these were generally discriminatory in terms of time periods.

Another issue may arise from participation rates in the study. Although rates were similar or better than those in similar population-based studies, they were imperfect, with a lower response among controls. This might have influenced the socioeconomic profile of participants. To evaluate the potential for selection bias into the study, we conducted analyses comparing study participants and non-participants using four ecological variables derived from census tract data for 2006. The percentages of subjects living in areas with a greater proportion of recent immigrants within the previous 5 years were 5 and 6%, for participants and non-participants, respectively. Corresponding values were 7 and 7% for higher unemployment rate, 20 and 21% of adults without a high school diploma, and 23 and 25% in the lowest quintile of household income, suggesting a slight trend toward more advantageous SEP among participants. This held true in analyses by case/control status. Based on these observations, it appears that there was no major selection bias in our study. Characteristics on non-participants are rarely collected in epidemiologic studies, leaving uncertainties on potentially inherent selection biases that might have occurred in previous investigations.

Advantages of the study include its relatively large sample, the quality of job history information, the inclusion of different indicators of SEP at three points in time and the detailed data collection enabling the consideration of several co-factors, including PCa screening, as well as the ability to take into account cancer aggressiveness. Finally, we used a novel Bayesian approach for investigating life course models, which allowed us to estimate the probability that the data supports the models.

## Conclusion

Our study examined the association between life course SEP and the risk of PCa. Overall, our findings provide evidence for periods in life which are more sensitive to exposure to disadvantageous SEP in relation to PCa risk. Late adulthood was consistently found to be a sensitive period to disadvantageous SEP exposure in relation to risk of aggressive PCa. An association between SEP during childhood/adolescence and non-aggressive PCa was also observed, but based on only one of the SEP indicators used. Our findings require replication and warrant for more detailed exploration into the mechanisms through which disadvantageous SEP affects PCa risk during different exposure periods. From a prevention perspective, these results provide a valuable starting point for future research and suggest periods when intervention may be more beneficial.

**Table:** 

**What is already known on this subject?**
The role of socioeconomic position in prostate cancer development remains debated. Most previous studies have relied on one or two indicators of socioeconomic position assessed during adulthood, which may not have captured the whole exposure period or changes over the lifetime.
**What this study adds?**
The present study builds on a more comprehensive assessment of socioeconomic position using the life course approach measuring socioeconomic position from childhood to adulthood. The study provides evidence for a differential role of socioeconomic position in prostate cancer aggressiveness depending on the period of exposure.

## Ethics statement

Ethics committees of Maisonneuve-Rosemont Hospital, Jean-Talon Hospital, Charles-Lemoyne Hospital, Centre hospitalier Fleury, Institut National de la Recherche Scientifique (INRS), and the Ethics Committee for Health Research of the Université de Montréal approved the study. All participants provided written informed consent.

## Author contributions

SM and CB (equal contribution) conceived the conceptual framework and co-wrote the draft manuscript. SM performed the data analysis. BN contributed to the design and analysis strategy of the study. HR prepared the databases and contributed to the analyses. M-ÉP conceived and conducted the main PROtEuS study. All authors read, commented and approved the final manuscript.

### Conflict of interest statement

The authors declare that the research was conducted in the absence of any commercial or financial relationships that could be construed as a potential conflict of interest.
