# Supplementary material for: Disadvantageous Socioeconomic Position at Specific Life Periods May Contribute to Prostate Cancer Risk and Aggressiveness
Source: Front Oncol. 2018 Nov 15;8:515. doi: 10.3389/fonc.2018.00515 (PMC6249302; doi:10.3389/fonc.2018.00515)
Supplement: Supplementary file 1 [file Table_1.DOCX]

**Supplementary Material**

**TABLE 1. Life period specific odds ratios and 95% credible intervals for the association between a disadvantageous socio-economic position and prostate cancer risk, using parents’ ownership of a car as indicator variable for socioeconomic position during childhood and adolescence**

|  | **Regardless of screening** | | |  | | | **Restricted to screened within 2 years** | | | | | |
| --- | --- | --- | --- | --- | --- | --- | --- | --- | --- | --- | --- | --- |
| **All prostate cancers** |  |  |  | | |  |  | |  | |  |  |
|  | **Controls**  **(n=1883)** | **Cases**  **(n=1785)** | **Model 1*^a^***  **OR (95% Crl)** | | **Model 2*^b^***  **OR (95% Crl)** | | **Controls**  **(n=1438)** | **Cases**  **(n=1773)** | | **Model 1*^a^***  **OR (95% Crl)** | | **Model 2*^b^***  **OR (95% Crl)** |
| Childhood & adolescence | 896 (47.6) | 852 (47.7) | 1.23  (1.04 – 1.43) | | 1.20  (1.02 – 1.41) | | 677 (47.1) | 845 (47.7) | | 1.30  (1.10 – 1.52) | | 1.26  (1.06 – 1.48) |
| Early-adulthood | 1119 (59.4) | 1069 (59.9) | 1.03  (1.00 – 1.12) | | 1.03  (1.00 – 1.12) | | 839 (58.3) | 1064 (60.0) | | 1.05  (1.00 – 1.16) | | 1.05  (1.00 – 1.16) |
| Late-adulthood | 895 (47.5) | 881 (49.4) | 1.07  (1.00 – 1.19) | | 1.06  (1.00 – 1.19) | | 657 (45.7) | 876 (49.4) | | 1.15  (1.01 – 1.33) | | 1.12  (1.01 – 1.30) |
| **Non-aggressive prostate cancers** | |  |  | |  | |  |  | |  | |  |
|  | **Controls**  **(n=1883)** | **Cases**  **(n=1292)** |  | |  | | **Controls**  **(n=1438)** | **Cases**  **(n=1282)** | |  | |  |
| Childhood & adolescence | 896 (47.6) | 618 (47.8) | 1.27  (1.04 – 1.51) | | 1.27  (1.04 – 1.51) | | 677 (47.1) | 612 (47.7) | | 1.36  (1.13 – 1.62) | | 1.32  (1.08 – 1.58) |
| Early-adulthood | 1119 (59.4) | 754 (58.4) | 1.03  (1.00 – 1.11) | | 1.03  (1.00 – 1.11) | | 839 (58.3) | 750 (58.5) | | 1.05  (1.00 – 1.15) | | 1.05  (1.00 – 1.16) |
| Late-adulthood | 895 (47.5) | 608 (47.1) | 1.04  (1.00 – 1.13) | | 1.04  (1.00 – 1.13) | | 657 (45.7) | 604 (47.1) | | 1.07  (1.00 – 1.22) | | 1.07  (1.00 – 1.22) |
| **Aggressive prostate cancers** | |  |  | |  | |  |  | |  | |  |
|  | **Controls**  **(n=1883)** | **Cases**  **(n=491)** |  | |  | | **Controls**  **(n=1438)** | **Cases**  **(n=489)** | |  | |  |
| Childhood & adolescence | 896 (47.6) | 233 (47.5) | 1.09  (1.00 – 1.29) | | 1.07  (1.00 - 1.25) | | 677 (47.1) | 232 (47.4) | | 1.12  (1.00 – 1.34) | | 1.10  (1.00 – 1.31) |
| Early-adulthood | 1119 (59.4) | 313 (63.7) | 1.07  (1.00 – 1.24) | | 1.06  (1.00 - 1.22) | | 839 (58.3) | 312 (63.8) | | 1.09  (1.00 – 1.29) | | 1.08  (1.00 – 1.27) |
| Late-adulthood | 895 (47.5) | 272 (55.4) | 1.24  (1.02 – 1.54) | | 1.17  (1.00 - 1.46) | | 657 (45.7) | 271 (55.4) | | 1.39  (1.10 – 1.73) | | 1.28  (1.02 – 1.63) |

***^a^*** Model 1 – adjusted for age, ancestry, family history of prostate cancer.

*^b^* Model 2 – further adjusted for body mass index, physical activity, cigarette smoking, and alcohol drinking.

TABLE 2. Association between a disadvantageous socioeconomic position and prostate cancer risk, using the father’s longest occupation as indicator variable for socioeconomic position during childhood and adolescence

|  | **Controls**  **n (%)** | **Cases**  **n (%)** | **OR (95% Crl)*^a^*** | **Mean weight (95% Crl)** |
| --- | --- | --- | --- | --- |
| **Any type of PCa** | **(n=1399)** | **(n=1740)** |  |  |
| **Overall effects** |  |  | 1.27 (1.02 - 1.56) |  |
| **Weights** |  |  |  |  |
| Childhood & adolescence (w1) | 675 (48.2) | 896 (51.5) |  | 0.27 (0.01 - 0.69) |
| Early Adulthood (w2) | 812 (58.0) | 1040 (59.8) |  | 0.20 (0.01 - 0.65) |
| Late Adulthood (w3) | 631 (45.1) | 853 (49.0) |  | 0.52 (0.06 - 0.90) |
|  | | | | |
| **Non-aggressive PCa** | **(n=1399)** | **(n=1255)** |  |  |
| **Overall effects** |  |  | 1.12 (0.90 - 1.41) |  |
| **Weights** |  |  |  |  |
| Childhood & adolescence (w1) | 675 (48.2) | 634 (50.5) |  | 0.33 (0.02 - 0.80) |
| Early Adulthood (w2) | 812 (58.0) | 730 (58.2) |  | 0.28 (0.01 - 0.77) |
| Late Adulthood (w3) | 631 (45.1) | 586 (46.7) |  | 0.39 (0.02 - 0.85) |
|  | | | | |
| **Aggressive PCa** | **(n=1399)** | **(n=483)** |  |  |
| **Overall effects** |  |  | 1.61 (1.20 - 2.18) |  |
| **Weights** |  |  |  |  |
| Childhood & adolescence (w1) | 675 (48.2) | 261 (54.0) |  | 0.24 (0.01 - 0.59) |
| Early Adulthood (w2) | 812 (58.0) | 308 (63.8) |  | 0.18 (0.01 - 0.54) |
| Late Adulthood (w3) | 631 (45.1) | 266 (55.1) |  | 0.58 (0.16 - 0.91) |
|  | | | | |

*^a^* Model adjusted for age, ancestry, family history of prostate cancer, body mass index, physical activity, cigarette smoking, and alcohol drinking

**
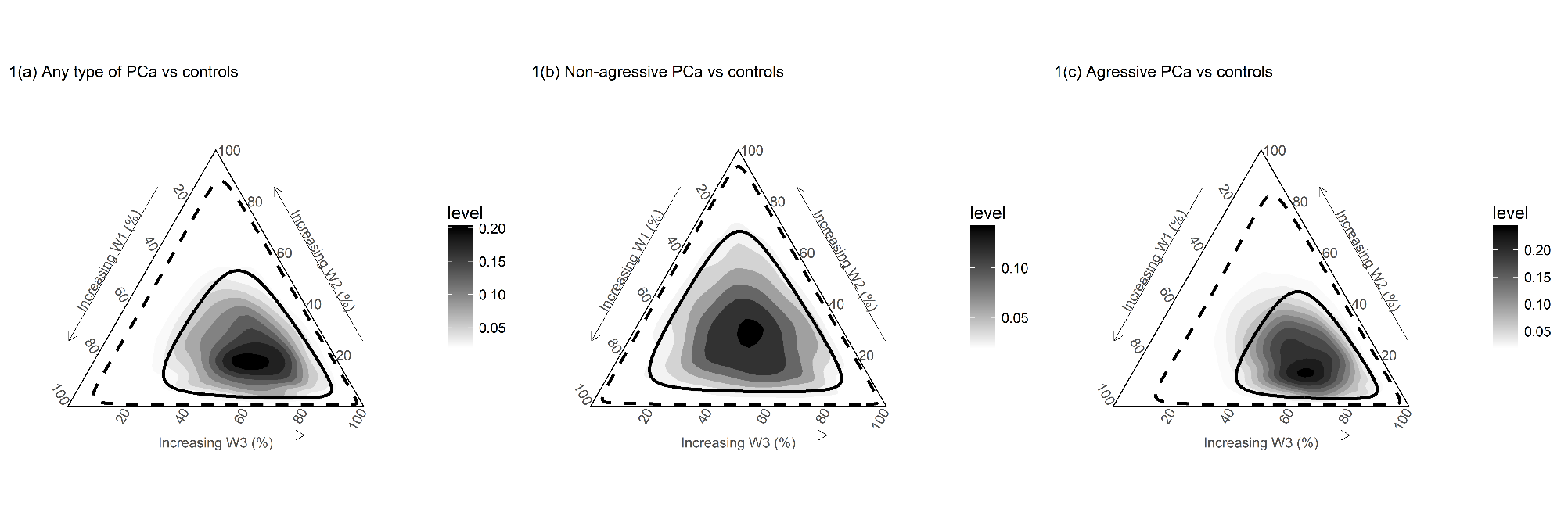
**

FIGURE 1S. Posterior joint distribution of weights estimated for three periods (childhood & adolescence based on father’s longest occupation [w1], early adulthood [w2], late adulthood [w3]). 2(a) All PCa cases vs control. 2(b) Non-aggressive PCa cases (Gleason <7) vs controls. 2(c) Aggressive PCa cases (Gleason>=7) vs controls. Solid and dashed line represents 50% and 95% credible intervals and darker areas represent higher posterior densities.

**TABLE 3. Life period specific odds ratios and 95% credible intervals for the association between a disadvantageous socioeconomic position and prostate cancer risk, using the father’s longest occupation as indicator variable for socioeconomic position during childhood and adolescence**

|  | **Regardless of screening** | | | | | | **Restricted to screened within 2 years** | | | |
| --- | --- | --- | --- | --- | --- | --- | --- | --- | --- | --- |
| **All PCa cases** | |  |  | |  |  |  |  |  |  |
|  | | **Controls**  **(n=1829)** | **Cases**  **(n=1751)** | **Model 1*^a^* OR (95% Crl)** | | **Model 2*^b^***  **OR (95% Crl)** | **Controls**  **(n=1399)** | **Cases**  **(n=1740)** | **Model 1*^a^***  **OR (95% Crl)** | **Model 2*^b^***  **OR (95% Crl)** |
| Childhood & Adolescence | | 908 (49.6) | 901 (51.5) | 1.02  (0.96 – 1.12) | | 1.02  (0.95 – 1.11) | 675 (48.2) | 896 (51.5) | 1.07  (1.00 – 1.19) | 1.07  (1.00 – 1.21) |
| Early-Adulthood | | 1080 (59.0) | 1045 (59.7) | 1.02  (0.96 – 1.11) | | 1.02  (0.94 – 1.10) | 812 (58.0) | 1040 (59.8) | 1.05  (1.00 – 1.16) | 1.05  (1.00 – 1.16) |
| Late-Adulthood | | 859 (47.0) | 858 (49.0) | 1.05  (0.98 – 1.19) | | 1.04  (0.96 – 1.18) | 631 (45.1) | 853 (49.0) | 1.18  (1.01 – 1.37) | 1.14  (1.00 - 1.34) |
| **Non-aggressive PCa** | | |  |  | |  |  |  |  |  |
|  | | **Controls**  **(n=1829)** | **Cases**  **(n=1264)** |  | |  | **Controls**  **(n=1399)** | **Cases**  **(n=1255)** |  |  |
| Childhood & Adolescence | | 908 (49.6) | 637 (50.4) | 0.99  (0.89 – 1.06) | | 0.99  (0.89 – 1.07) | 675 (48.2) | 634 (50.5) | 1.04  (0.97 – 1.17) | 1.04  (0.96 – 1.16) |
| Early-Adulthood | | 1080 (59.0) | 734 (58.1) | 0.99  (0.89 – 1.06) | | 0.99  (0.89 – 1.06) | 812 (58.0) | 730 (58.2) | 1.03  (0.98 – 1.14) | 1.03  (0.96 – 1.13) |
| Late-Adulthood | | 859 (47.0) | 590 (46.7) | 0.99  (0.92 – 1.08) | | 0.99  (0.91 – 1.09) | 631 (45.1) | 586 (46.7) | 1.06  (0.98 – 1.22) | 1.05  (0.97 – 1.22) |
| **Aggressive PCa** | | |  |  | |  |  |  |  |  |
|  | | **Controls**  **(n=1829)** | **Cases**  **(n=485)** |  | |  | **Controls**  **(n=1399)** | **Cases**  **(n=483)** |  |  |
| Childhood & Adolescence | | 908 (49.6) | 263 (54.2) | 1.09  (1.00 – 1.29) | | 1.08  (1.00 – 1.28) | 675 (48.2) | 261 (54.0) | 1.13  (1.01 – 1.36) | 1.31  (1.00 – 1.36) |
| Early-Adulthood | | 1080 (59.0) | 309 (63.7) | 1.08  (1.00 – 1.26) | | 1.07  (1.00 – 1.24) | 812 (58.0) | 308 (63.8) | 1.09  (1.00 – 1.30) | 1.09  (1.00 – 1.28) |
| Late-Adulthood | | 859 (47.0) | 267 (55.1) | 1.25  (1.02 – 1.56) | | 1.19  (1.00 – 1.49) | 631 (45.1) | 266 (55.1) | 1.41  (1.11 – 1.76) | 1.32  (1.04 – 1.69) |

***^a^*** Model 1 – adjusted for age, ancestry, family history of prostate cancer.

***^b^*** Model 2 – further adjusted for body mass index, physical activity, cigarette smoking, and alcohol drinking.

TABLE 4. Correlation between different measures of socioeconomic position across the life course among control participants *^a^*

|  | Parents’ ownership of a car | Father’s longest occupation | Participant’s first occupation | Participant’s longest occupation |
| --- | --- | --- | --- | --- |
| Parents’ ownership of a car | 1 | (n=1910) | (n = 1981) | (n = 1981) |
| Father’s longest occupation | 0.06 | 1 | (n=1916) | (n=1916) |
| Participant’s first occupation | 0.10 | 0.20 | 1 | (n=1991) |
| Participant’s longest occupation | 0.18 | 0.16 | 0.55 | 1 |

*^a^* Lower diagonal represents the Spearman’s correlation coefficients and the upper diagonal represents pairwise sample sizes.
